# Long-Term Outcomes of a Novel Surgical Approach for Early Charcot Neuroarthropathy

**DOI:** 10.1155/cro/3342675

**Published:** 2025-10-29

**Authors:** Andrew Rader, Aaron Ruter, Colby Holmes, Alyssa Zacharjasz

**Affiliations:** ^1^Private Practice, Indiana Foot & Ankle, Jasper, Indiana, USA; ^2^Orthopedics Department, Lakeshore Bone and Joint, Crown Point, Indiana, USA; ^3^Surgery Department, St. Claire Healthcare, Morehead, Kentucky, USA; ^4^Surgery Department, Mahaska Health, Oskaloosa, Iowa, USA

## Abstract

**Background:**

Charcot neuroarthropathy (CN) of the foot is a progressive condition associated with significant deformity and recurrent ulceration. Despite advancements in imaging and classification, management remains challenging. Traditional nonsurgical treatments have shown limited success in preventing disease progression, while surgical interventions often involve prolonged nonweight-bearing periods and high complication rates.

**Methods:**

This study presents an 8-year follow-up of 15 patients (Evidence Level IV) undergoing a novel early surgical intervention for Stage 0-1 CN with preoperative plantigrade alignment. The procedure involved fluoroscopically guided filling of subchondral defects using a flowable calcium phosphate compound and realignment with dynamic circular external fixation in 14 of 15 subjects. Assisted weight-bearing began 3–5 days postoperatively, with fixation removed after 6–8 weeks. Radiographic parameters (Meary's angle, calcaneal inclination angle, talar declination angle, and cuboid height) were assessed preoperatively and at final follow-up.

**Results:**

Thirteen of 15 subjects survived the 8 years. No surgical complications, ipsilateral CN, or midfoot ulcerations occurred. Radiographic alignment remained stable over 8 years (*p* < 0.01 for all parameters).

**Conclusion:**

Early surgical intervention with subchondral defect filling and external fixation appears to stabilize osseous architecture, prevent midfoot collapse, and expedite recovery in CN. Larger prospective studies are warranted to validate these findings.

## 1. Introduction

Charcot neuroarthropathy (CN) is a debilitating complication of neuropathy that leads to progressive deformity and ulceration [1]. The annual incidence of CN in patients with diabetes is estimated to be 27,602/year, with a prevalence of more than 200,000 in the United States [2]. This exceeds the incidence of many cancers [3]. Early recognition is critical, as intervention before collapse can preserve foot structure.

Clinically, early CN often presents with unilateral warmth, swelling, and erythema. A temperature difference greater than 2°C compared to the contralateral limb is suggestive. Differential diagnoses include infection, gout, and DVT. Radiographically, plain x-rays are frequently normal in early development, but MRI is highly sensitive, demonstrating bone marrow edema, microfractures, and effusions. Weight-bearing CT allows earlier detection of subtle collapse and accurate measurement of Meary's angle, calcaneal inclination, and cuboid height [4, 5].

The Eichenholtz classification system was described in the 1960s (development, coalescence, and reconstruction) [6]. Subsequently, these findings have been confirmed with the advent of advanced imaging technologies. As a result of improved imaging, an earlier Stage 0 was added to the previous three Eichenholtz stages. This stage is characterized by bone edema, stress fractures, soft tissue edema, cartilage damage, joint effusion, and subluxations [7].

The pathophysiology of CN is still being discovered; however, several observations have been made. Initially, an unabated inflammation is present [8]. This triggers an overproduction of RANKL, an important mediator of osteoclastogenesis [9, 10]. In CN, there is observed a disbalance of osteoclasts to osteoblasts. This disbalance has been implicated in observed decreased bone mineral density in the active CN foot [11].

The resultant demineralized bone results in unique challenges in the management of CN. Historically, nonsurgical treatment has been promoted as the standard of care [12]. Unfortunately, ulceration rates of 67%–75% are reported, and the progressive nature of the disease has been implicated [13]. When surgical treatment, regardless of the method, is used, the mean time to weight-bearing is 17 weeks. The prolonged period of nonweight-bearing also profoundly affects the bone density. Complication rates for surgical reconstruction are reported to be 36% [14], but this is not addressing long-term complications such as loss of osseous architecture, hardware failure, recurrent ulcerations, or shortened life span. Long-term data for either nonoperative or operative care of CN is sparse.

We conducted an 8-year follow-up of a case series involving 15 patients who underwent a novel early surgical intervention for CN. This technique has not been previously published. The goal of this case series was to investigate whether this novel intervention might preserve osseous structure, reduce recurrence, and maintain function.

## 2. Methods

An 8-year follow-up of a 15-subject case series (Evidence Level IV, case series) employing a novel early surgical intervention for CN was performed using existing chart records and phone follow-up for patients no longer present in the geographic area of our practice. Our practice is located in rural Midwestern United States, and the race is primarily Caucasian. Patient demographics are listed in Table 1.

Institutional review board approval was obtained from Memorial Hospital and Healthcare Center, Jasper, Indiana, United States, in 2015 for a feasibility study to be performed in a prospective fashion. Written informed consent for procedures, photographs, and patient information was obtained from all subjects.

### 2.1. Inclusion Criteria

• Diagnosis of CN is determined by a multiplicity of signs and symptoms including edema and warmth.
• MRI findings of bone marrow edema in multiple midfoot bones consistent with CN.
• Eichenholtz Stage 0-1 was included, but the foot had to be plantigrade preoperatively. Plantigrade is defined by the heel and forefoot touching the ground during stance which precludes the presence of a rocker-bottom foot deformity from advanced CN midfoot destruction.

### 2.2. Exclusion Criteria

• Active infection is determined by complete blood cell count, erythrocyte sedimentation rate, and C-reactive protein laboratory examination.
• Ankle-brachial index < 0.6.

### 2.3. Surgical Technique

**Figure 1:**
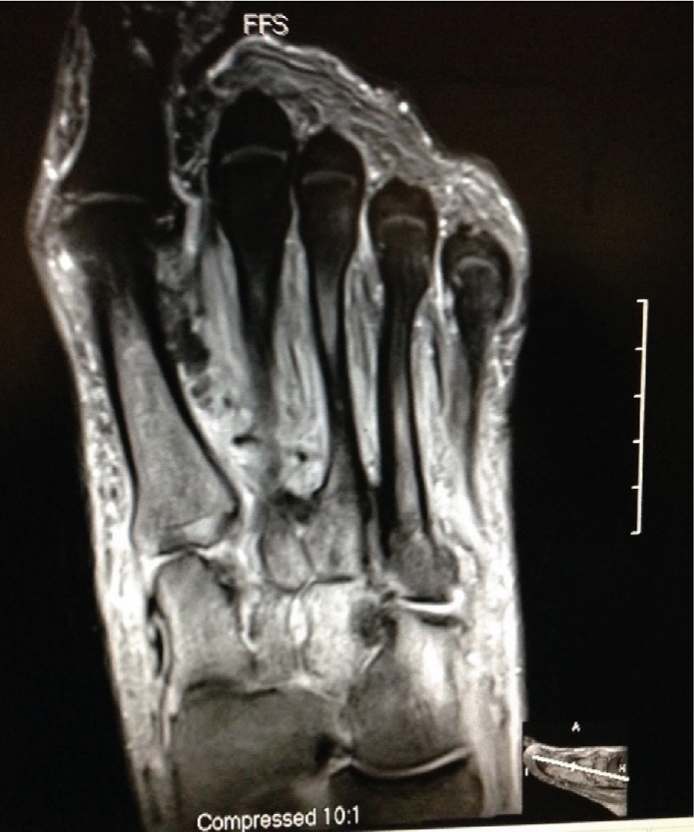
T2-weighted MRI of an example Charcot foot that demonstrates bone edema in a multitude of tarsal–metatarsal bones.

**Figure 2:**
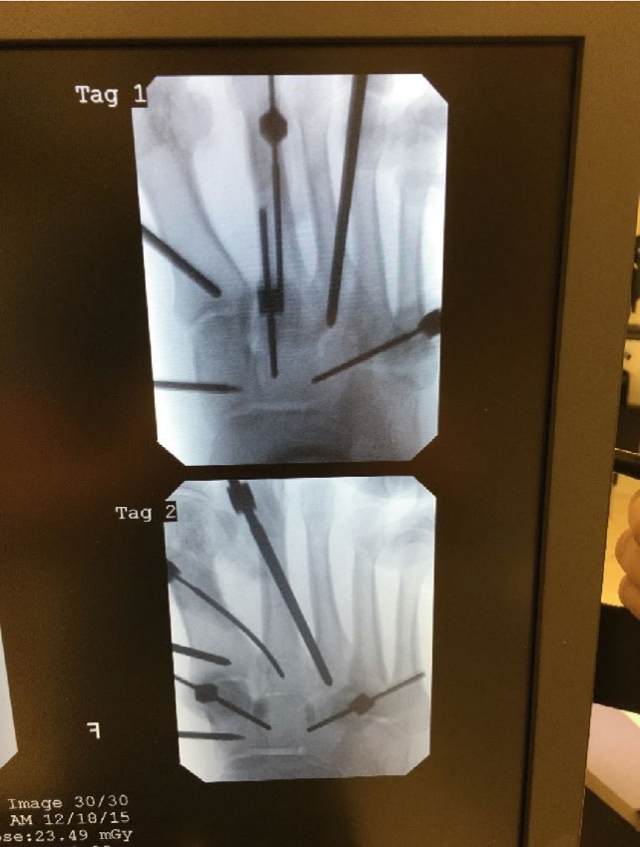
Intraoperative fluoroscopically guided filling of subchondral defects with engineered calcium phosphate mineral compound.

**Figure 3:**
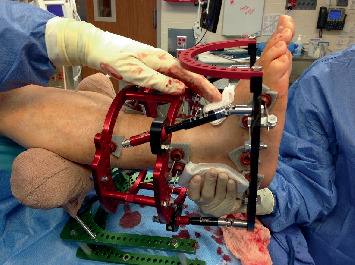
Example of dynamic circular external fixation used to reduce subluxations and maintain alignment and stability during the postoperative period.

Fifteen subjects underwent fluoroscopically guided filling of subchondral defects in all bones demonstrating edema on MRI (Figure 1). The product (Figure 2) used was a flowable engineered calcium phosphate mineral compound [15]. Subluxations were realigned in 14 of the 15 subjects with dynamic circular external fixation utilizing a bent wire technique (Figure 3). Assisted weight-bearing began at 3–5 days postoperatively (PO). Gastrocnemius recession was performed on all the patients. One subject utilized a Charcot restraint orthotic walking (CROW) boot only (PO). The external fixation was left in place for 6 weeks for 12 subjects and 8 weeks for 2 others.

**Figure 4:**
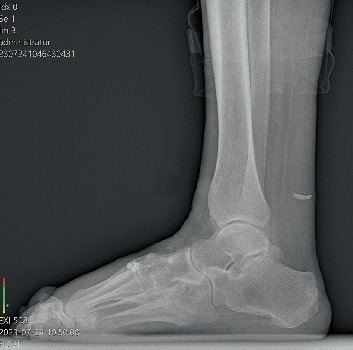
Eight-year postoperative x-ray demonstrating retained anatomic alignment.

The patients were followed long term with yearly x-rays (Figure 4) and/or weight-bearing CT scans. Preoperative and PO radiographs were measured to assess midfoot collapse. Radiographic measurements include lateral talo-first metatarsal angle (Meary's), calcaneal inclination angle (CIA), talar declination angle (TDA), and cuboid height. Normal measurement ranges were obtained from the literature as follows: Meary's 0°–15°, CIA 8.5°–30°, and TDA 14°–30° [16]. These values were used to determine a hypothetical mean. Using an online statistical calculator program, a one-sample *t*-test was utilized for preoperative and 8-year PO radiographic measurements (Table 1). One patient with acute CN of the ankle was excluded from these final measurements.

## 3. Results (Table 2)

At 8 years PO, 13 of 15 patients were alive. Two deaths (one cardiac, one accidental fall) were unrelated to the procedure. No surgical complications were observed. No recurrence of ipsilateral CN occurred, although one patient developed CN in the contralateral foot 5 years PO. No midfoot ulcerations developed.

Radiographic outcomes demonstrated stable alignment across all parameters. Meary's angle averaged 16° at baseline and remained unchanged at follow-up (*p* = 0.0007). CIA increased slightly from 21.8° to 22.1° (*p* = 0.0001), and TDA decreased from 17.5° to 17.2° (*p* = 0.0013). Inclusion of final radiographs from deceased patients did not alter statistical significance. Comparative results are presented in Table 2.

## 4. Discussion

Conventional nonoperative care for early CN includes prolonged total contact casting [11], yet progression to deformity and ulceration remains common. Operative reconstruction often delays weight-bearing for more than 17 weeks and carries high complication rates [13].

In this case series, patients achieved assisted ambulation within 3–5 days and transitioned to shoe gear by 8–10 weeks. By 12 weeks PO, all patients were allowed to return to usual activities, including return to work. Radiographic stability was preserved.

Calcium phosphate augmentation was selected for its role as a biomechanical buttress, filling subchondral defects vulnerable to fracture. Although it has no direct biochemical effect on RANKL-mediated osteoclastogenesis, it functions as an osteoconductive scaffold. Limiting inclusion criteria to plantigrade feet avoided complex reconstructions, targeting cases where anatomy could be preserved. Limitations include a small sample size and absence of validated functional scores. Nonetheless, return to work and activities of daily living suggest a positive quality-of-life impact. Prospective studies using validated scales are recommended. In conclusion, early surgical stabilization with calcium phosphate and external fixation may reduce ulceration risk, preserve alignment, and enable earlier functional recovery in early CN.

## Figures and Tables

**Table 1:** Patient demographics and baseline clinical data.

**Variable**	**n** = 15
Mean age, years (range)	54.8 (35–69)
Sex, male/female	9/6
Diabetes Type 1/Type 2	0/15
Mean duration of diabetes, years	14.5 (6–27)
Mean HbA1c (%)	7.9 (6.5–9.4)
Smoking history	4
Peripheral arterial disease (ABI < 0.9 but ≥ 0.6)	3
Prior foot ulceration	2
Prior amputation	0
Eichenholtz Stage 0/1	6/9
Plantigrade alignment (all patients)	15

**Table 2:** Radiographic outcomes (preoperative versus final follow-up).

**Parameter**	**Preoperative ( ** **m** **e** **a** **n** ± **SD, range)**	**Final follow-up ( ** **m** **e** **a** **n** ± **SD, range)**	**p** ** value**
Meary's angle (°)	16.4 ± 3.2 (12–21)	16.0 ± 3.1 (11–20)	0.0007
Calcaneal inclination angle (°)	21.8 ± 2.9 (18–26)	22.1 ± 3.1 (17–27)	0.0001
Talar declination angle (°)	17.5 ± 2.7 (13–22)	17.2 ± 2.8 (12–21)	0.0013
Cuboid height (mm)	14.1 ± 2.4 (10–18)	14.3 ± 2.6 (11–19)	NS

## Data Availability

The datasets generated and analyzed during the current study are available from the corresponding author on reasonable request.
